# Hand Trauma in Emergency Department Management in Older Adults ≥ 80 Years Old: A Twenty-Year Retrospective Analysis

**DOI:** 10.3390/geriatrics8060112

**Published:** 2023-11-15

**Authors:** Camillo Fulchignoni, Marcello Covino, Silvia Pietramala, Ivo Lopez, Gianfranco Merendi, Andrea De Matthaeis, Francesco Franceschi, Giulio Maccauro, Lorenzo Rocchi

**Affiliations:** 1 Orthopedics & Hand Surgery Unit, Department of Orthopedics, Fondazione Policlinico Universitario A. Gemelli, IRCCS, Università Cattolica del Sacro Cuore, 00168 Rome, Italy; silvia.pietramala01@gmail.com (S.P.); ivolopez2797@gmail.com (I.L.); gianfrancomerendi@icloud.com (G.M.); lorenzo.rocchi@policlinicogemelli.it (L.R.); 2 Department of Emergency Medicine, Fondazione Policlinico Universitario A. Gemelli, IRCCS, Università Cattolica del Sacro Cuore, 00168 Rome, Italy; macovino@gmail.com (M.C.); francesco.franceschi@policlinicogemelli.it (F.F.); 3 Orthopedics & Traumatology Unit, Department of Orthopedics, Fondazione Policlinico Universitario A. Gemelli, IRCCS, Università Cattolica del Sacro Cuore, 00168 Rome, Italy; andrea.dematthaeis@policlinicogemelli.it (A.D.M.); giulio.maccauro@policlinicogemelli.it (G.M.)

**Keywords:** hand surgery, elderly, associated injuries, emergency department, patient admission

## Abstract

The prevalence of hand injuries increases with age, with elderly patients being more prone to hand lesions due to a combination of factors, such as reduced bone density and muscle strength, impaired sensation, and cognitive impairment. Despite the high incidence of hand injuries in the elderly population, few studies have addressed the management and outcomes of hand lesions in this age group. This study aimed to analyze the characteristics and management of hand lesions in patients over 80 years old. The authors conducted a retrospective analysis of medical records of patients over 80 years old who reached their Emergency Department with hand lesions between 2001 and 2020. Data on demographics, injury characteristics, and management were collected and analyzed. A total of 991 patients with hand lesions were included in the study, with a mean age of 84.9 years. The most common causes of injuries were domestic accidents (32.6%) and traffic accidents (12.8%). The most frequent types of hand lesions were fractures (23.5%) and superficial wounds (20.5%). Overall, 23.4% underwent surgical treatment for their hand issue, and 22.1% had associated injuries, among which, the most common were head trauma and other bone fractures. In conclusion, hand lesions in patients over 80 years old are frequent and pose significant challenges in diagnosis and management. Particular attention should be paid to associated injuries and limit indications to surgery when strictly necessary.

## 1. Introduction

Approximately 20% of all emergency department (ED) admissions for injury in Italy are relative to upper extremity injuries [1]. Up to 60% of those, representing approximately 880,000 patients/year, are evaluated for a hand wound. Hand injuries are one of the most frequently encountered lesions treated in the ED. However, these numbers could be even higher since hand injuries are often neglected [2], particularly in severely traumatized patients.

With the aging of the world population in recent decades [3,4], health conditions and medical emergencies in older patients have gained great relevance [5,6]. Most research focuses on neurological [7,8], gastrointestinal [9], metabolic [10], plastic [11], and orthopedic issues [12,13,14] in older adults, but no authors have ever focused their work on hand trauma. In Giustini’s [1] epidemiological study on upper extremity injuries, the most commonly diagnosed injury in the over-80-year-old population is a “closed fracture of unspecified part of the upper end of humerus”, without any mention of hand trauma in this category of patients. Confronted with this lack of data, the authors wonder if elderlies really do not injure their hands.

To the authors’ knowledge, up to the present, there is no other article in the literature analyzing the epidemiology of hand trauma in very old adults (over 80 years old).

This article aimed to study hand lesions in patients ≥ 80 years in the ED from an epidemiological point of view. Particular attention was paid to the analysis of the characteristics of lesions and lesions’ mechanisms and the management of the elderly with hand trauma in the Emergency Department. To complete their study, the authors compared the obtained results to those in the literature regarding the general population.

## 2. Materials and Methods

The digital records of the patients admitted to the ED of our institution (Policlinico Agostino Gemelli, Rome, Italy) were retrospectively evaluated.

The authors searched for the keywords “hand” and “finger” on the discharge diagnosis available on their ED software (GIPSE^®^ version 6.0.0.8) for all patients over 80 years of age admitted between 1 January 2001 and 31 December 2020. All patients with carpal, metacarpal, and phalangeal injuries have been included, whereas patients with radius, ulna, and humeral injuries have been excluded. Four authors manually and independently reviewed the clinical records of the extracted patients. In the case of disagreement about whether to include a patient in this study, when consensus was not achieved, the first author took the final decision.

The information collected for each patient included:

- Age;
- Sex;
- Triage severity code (by trained triage nurse);
- If the patient underwent surgery for the hand lesion that brought them to the ED;
- If the patient was hospitalized directly from the ED (if so, was it in the Hand Surgery Ward?);
- Cause of the accident: domestic, work, traffic, other (including self-harm lesions, bites, assaults), or non-specified;
- Type of injury: amputation, fracture, dislocation, tendinous, wound, bruise/sprain, pain (arthritic, from nerve compression, or other), or “other” (including ischemia, burns, infection, foreign body, bites, and wound dressing);
- If there were any associated injuries (what and where?).

The results are first presented generally over the 20 years analyzed for epidemiological purposes, with ages reported as means, ranges, and standard deviations (SD), and other data are presented as a percentage. Secondly, the results are subdivided into five-year periods (2001–2005, 2006–2010, 2011–2015, 2016–2020) to analyze any eventual variation in time. The significance of the evolution of bounded categories (triage severity code, surgery, and hospitalization) was assessed by comparing their distribution using a two-sided chi-square test—using Microsoft Excel (2019)—with a confidence level of 95% and a significance level α = 0.05.

## 3. Results

### 3.1. 2001–2020

Over the 20 years analyzed, 991 over-80-year-old patients reached our ED with a hand injury (Table 1). Among these, 519 were female (52.4%), and 472 were male (47.6%). The mean age was 84.9 (85.3 for females, 84.6 for males).

Regarding the triage severity color code, among the 991 patients reviewed, 25 were coded as white (2.5%), 747 as green (75.4%), 208 as yellow (21.0%), and 11 as red (1.1%). The necessity of hand surgery can also be used as a hand lesion severity classification item. Of all the elderly patients with hand trauma who reached our ED during the 2001–2020 period, 232 patients (23,4%) underwent hand surgery.

Out of these 232 patients who needed surgery, 84 (36.2% of those who had surgery and 8.5% of all the patients who reached the ED with a hand problem) had been hospitalized directly from the ED, in opposition to those who had first been sent home and either were hospitalized at a later time or as outpatients. Furthermore, out of those 84 patients, 32 were hospitalized in a different ward from the Hand Surgery one because of their associated injuries.

Regarding the cause of the accident, for 402 patients (40.6%), this was not specified either because the patient did not communicate the cause or because they were not able to. Without taking into account those 402 patients for which the cause was not specified, most of injuries were domestic (323; 54.8%), followed by traffic accidents (127; 21.6%). Only five patients (0.8%) had their accident during work, and the remaining 134 were classified as “other”, which includes self-harm lesions, bites, and assaults.

The most common types of lesions were fractures, representing almost 25% of all lesions altogether. Among those, phalangeal fractures were most frequent, followed by metacarpal fractures. Carpal bone fractures were relatively rare (1.3%). Fractures were followed by superficial wounds, bruises, and sprains. Diffused hand pain was frequent, whereas amputations, tendon lesions, and dislocations represented approximately only 3% of lesions each (Table 2).

The highest numbers of surgeries performed were bone reduction and fixation srugeries (106 between phalanges and metacarpal bones), representing 51.7% of all metacarpal fractures and 38.2% of all phalangeal fractures; but lesions more frequently needing surgery were amputations (mostly to regularize the stump) and tendon lesions (to perform tendon sutures). Among the 35 patients who underwent surgery in the “others” group, 25 had an infection of the hand. Bruises and sprains, pain, and carpal fractures were never treated surgically in this series of patients.

**Figure 1:**
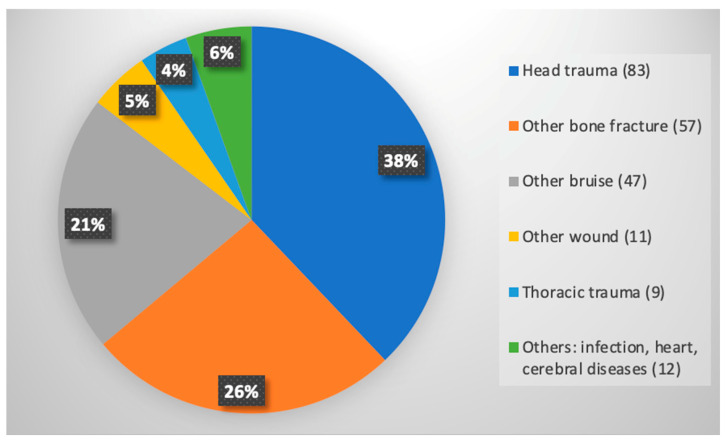
Associated injuries. Numbers in brackets represent absolute counts of patients with the specific type of associated injury; numbers in the chart represent percentages.

In older adults, it is not rare that hand trauma is initially neglected, especially when an associated injury is present [2]. In this study, more than one out of five patients had an associated injury: 219 (22.1%) out of 991 (Figure 1). The most frequent associated injury was “head trauma”, followed by “other bone fracture”, as the elderly are at higher risk of falling [15] and other bruises. Of these patients, only 32 needed hospitalizations in a different ward from the Hand Surgery one to treat their associated injuries.

### 3.2. Evolution over Time

**Figure 2:**
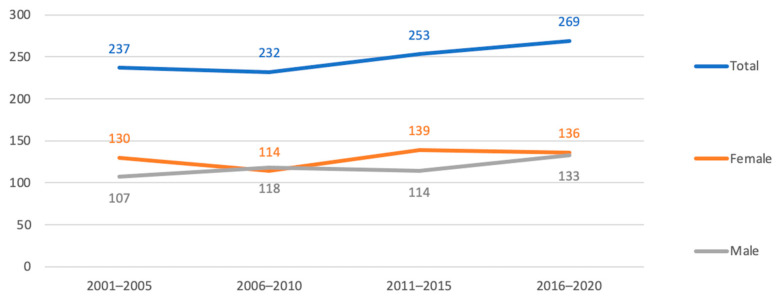
Evolution of the number of patients over 80 years old reaching the ED for a hand problem. Numbers in abscissae and in the graph represent the absolute number of patients; values in ordinates represent the period of time taken into account.

**Figure 3:**
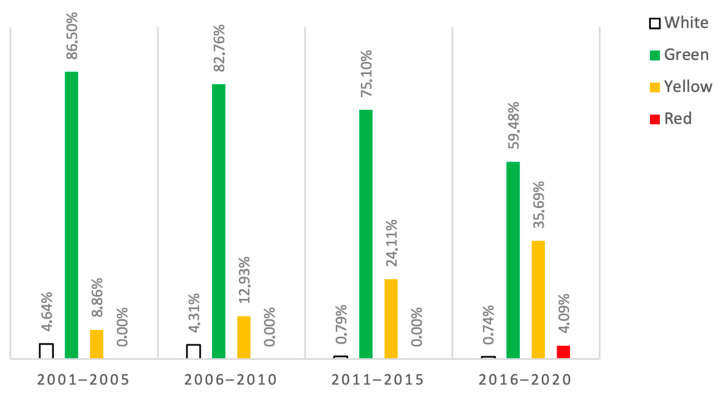
Evolution over time of severity of lesions (by triage code color) in percentages. White (not urgent, should be assessed within 240 min), green (minor urgency, should be assessed within 120 min), yellow (urgency, should be assessed within 30 min), and red (emergency, should be assessed immediately).

It appears that the number of patients over 80 years old seeking treatment in the Emergency Department due to hand injuries has slightly increased in recent decades, as revealed in Figure 2. From 2001–2005 to 2016–2020, the total number of patients over 80 years old treated in the ED for hand injuries went up from 237 to 269. Although the number of female patients remained relatively consistent, increasing only from 130 to 136, the number of male patients increased by 25%, from 107 in 2001–2005 to 133 in 2016–2020. The average age of patients did not vary significantly, remaining at 84.83 in 2001–2005 and 84.97 in 2016–2020 (*p* = 0.795). According to the color coding used in triage, the severity of hand injuries seems to have increased over the years. A statistically significant difference (*p* < 0.001) was found when comparing the distribution of patients in the white group (11 in 2001–2005 versus 2 in 2016–2020), green group (205 in 2001–2005 versus 160 in 2016–2020), yellow group (21 in 2001–2005 versus 96 in 2016–2020), and red group (0 in 2001–2005 versus 11 in 2016–2020) (Figure 3). However, this trend was not reflected in the percentage of patients requiring surgery, which decreased from 30.4% in 2001–2005 to 16.7% in 2016–2020, with a drop in between to 25% from 2006–2010 and 22.5% from 2011–2015.

## 4. Discussion

In recent years, due to population aging, hand surgeons, like other physicians, have become involved in treating elderly patients with changing medical needs [16,17,18,19]. Additionally, advancements in less invasive techniques and materials allow for the treatment of more delicate patients who may not be suitable for invasive surgeries [20,21].

The study found that there has been an increase in the number of patients aged 80 years or above with hand injuries in the emergency department over the last 20 years. This trend is likely due to the increase in the number of active elderly people in recent times [22]. The study also revealed that most of the injuries in this age group were a result of domestic accidents. Rosberg et al. [23] conducted a retrospective study of a similar population of individuals aged over 65 with hand injuries. Their analysis of four non-continuous two-year periods from 1980–1981 to 2010–2011 also revealed an increase in the number of hand injuries in the elderly population over the past three decades, with a growing number of injuries caused by domestic accidents.

The data from the study indicate that there has been a rise of around 10% in the number of patients aged 80 and above who have reported to the ED with hand injuries over the last 20 years. However, if we consider that the population of people aged 80 and above in Italy has doubled in the same period (from 2.3 million in 2000 to 4.5 million in 2020) [24], it appears that a smaller proportion of elderly people are actually reporting to the ED for hand injuries. On the other hand, the study shows that hand injuries among the elderly have been becoming more severe in recent years. The authors of the study believe that this could be due to the Italian government’s efforts to encourage people to visit their general practitioners for less serious injuries in the last few decades.

Fractures were the most common injuries, followed by superficial wounds or cuts without underlying lesions. Phalangeal and metacarpal reductions and fixations were the most frequently performed surgeries. As expected, tendon lesions and amputations were the injuries that required surgery most often. Some characteristics of the elderly population can easily explain the incidence of these injury causes, such as increasing osteoporosis [25], a higher risk of falls due to neurological pathologies [26], and a lower skin quality [27,28].

It is worth comparing the data presented here to the general population data that were gathered in a previous study by the same authors [29]. The previous study discussed the epidemiology of hand trauma before and after the COVID outbreak. According to that study, the average number of patients visiting the ED per year for hand trauma in the three years prior to the COVID pandemic was 1835. Therefore, the average of 50 patients over 80 years old seen per year represents less than 3% of the total. In the previous study, males were found to be more commonly involved in hand trauma (64.9%), whereas in this study, the ratio was almost 50–50, probably due to the longer life expectancy of women. The severity of injuries, classified by triage color code, seems to be comparable in both the general population and elderly population. In both studies, the most common cause of injury is “domestic accidents”, accounting for 22.1% in the general population and 32.6% in the elderly. Furthermore, among the general population, 8.6% of injuries are work-related, 4.8% are school-related, and 9.2% are sports-related. These types of injuries almost completely disappear in the elderly population due to obvious reasons. With regard to the type of injuries, while the elderly seem to be affected more often by fractures and less often by amputation and tendon lesions, these differences are not statistically significant. Finally, the data regarding the indication for surgery and hospitalization rates show that the elderly are more frequently treated conservatively (with a surgery rate of 23.4% versus 32.0% in the general population) and are more likely to be hospitalized when surgery is required (with a rate of 36.2% versus 26.7% in the general population).

There is a significant difference between the elderly population and the general population in terms of the frequency of associated injuries. Specifically, the over-80-years-old population tends to have a higher rate of such injuries. This can be attributed to various factors, such as increased fragility, a higher number of risk factors, and a slower reaction time to using their hands as a defensive tool while falling [30].

In recent years, despite an increase in triage severity, the number of patients requiring hand surgery has decreased. This can be attributed to the fact that from 2001 to 2015, most patients requiring hospitalization were admitted to a Hand Surgery ward, while in the last five years, only 7 were admitted to that ward out of 20 hospitalized patients. The remaining 13 were hospitalized in a different ward, indicating that the severity of the color code was likely due to associated injuries rather than just hand trauma.

To the best of the authors’ knowledge, only one other study has examined hand injuries in an elderly population [31]. In that study, all patients over 65 years old were included, but less than 20% were over 80 years old. Kringstad et al. also found that fractures were the most frequent injuries, followed by wounds and dislocations. Associated injuries were less frequent (13%), with head injuries being the most common. The lesions occurred outdoors in 33% of cases, indoors in 23%, at work in 4%, and in traffic incidents in 4%. In this study, a higher percentage of patients underwent surgery (36.6%), highlighting the need for a more conservative approach in older patients.

In 2019, Reitan et al. [32] conducted a retrospective study to evaluate the impact of upper limb injuries on the quality of life and hand disability in patients aged over 65. The study did not consider the type or cause of injury. The authors used various health-related questionnaires, including SF-36, the Cold Intolerance Severity Score, and Quick DASH. The results showed that patients reported limited functional impairment after upper distal upper limb injuries. However, those with a Cold Intolerance Severity Score over 50 were more severely affected.

In published epidemiological studies, many authors have examined hand injuries in the general population. From these studies, it is possible to extrapolate some interesting information about the elderly. In 2023, Moellhoff et al. [33] analyzed a ten-month period between 2015 and 2016, which included 435 patients reaching a tertiary care facility in Germany with a hand injury. Among them, 290 were male and 140 were female, with a mean age of 39.46 years old. When concentrating on the older population (70–90 years old), the ratio changed as only 7% of the total male population and 14% of the total female population were over 70. Two similar studies were published in 2021 using a national database to make whole-population estimations in the USA [34] and The Netherlands [35]. In the American study [34], the authors used the National Electronic Injury Surveillance System to evaluate 649,131 patients with hand injuries in involved centers over a ten-year period (2009–2018), which allowed them to estimate a total of 25,666,996 patients nationally. Among them, male patients represented 60% of cases, and females represented 40%. The mean age was 35.6 years old, fingers were more frequently involved than the hand, lacerations were the most frequent lesions followed by fractures and sprains, and male adults (18–39 years old) were the most frequent population. Analyzing the evolution over time, one of the author’s conclusions was that in patients 50 years of age and older, hand, wrist, and finger injuries increased over time. In the Dutch study, von Leerdam et al. [35] used a similar system, the Dutch Injury Surveillance System, to overview 19,324 patients treated in 2016 for hand injuries, leading to an estimation of 160,250 patients in the whole Dutch population. Among them, males accounted for 57% and females 43%, with a mean age of 33 years old. Fractures represented 55% of all lesions, and hand injuries accounted for 25% of all injuries that reached the Emergency Department. These authors noted that the incidence was higher among the male population younger than 55 years old, whereas the incidence was higher in females over 55 years old. Some of these data differ from those described by Arroyo-Berezowski et al. [36]. They studied 2184 patients with hand injuries reaching a specialized center in Mexico in 2015 with a hand or wrist issue. These patients represented only 9% of all patients reaching the Emergency Department. Fractures were still the most frequent type of injuries but represented only 34% of all trauma. The mean age was 31.85 years old, and the elderly population was only 0.9% of the whole population, with only 20 patients being over 80 years old. Finally, in an older study, Davas Aksan et al. [37] analyzed patients reaching a Hand and Microsurgery hospital in Izmir (Turkey) from 1992 to 2005, with quite different results. The mean age was 29.8 years old, and male patients represented 83% of the whole population. Most frequent lesions were amputations (32.5%) over fractures (23.7%) and open wounds (19.9%). The elderly population (over 65 years old) represented only 3% of the total, and among them, over 10% of the whole female population (156 among 1499) and only 1.5% of the male population (111 among 7404) were included. One of the author’s conclusions was that “the riskiest activity for hand injury was paid work”, which might explain why the elderly represented only a small portion of that population.

It is interesting to note that wrist injuries were included along with hand injuries in all of the aforementioned studies, which indicates a lack of attention toward the over-80-year-old population. It is well established that distal radius fractures are more common among the elderly due to osteoporosis, making these the most frequent upper limb injury in this age group [38]. O’Neil et al. [39] have demonstrated that the incidence of distal forearm fractures is highest in older populations, with 11 per 10,000 person-years in the 80–84-year-old male population, 23.3 per 10,000 in the over-85-year-old male population, 89.6 per 10,000 in the 80–84-year-old female population, and 116.8 per 10,000 in the over-85-year-old female population. Since literature is already available on this topic [40], the authors of this manuscript decided not to include distal radius fractures in their study.

Moreover, the review article by Alsawadi and Stanton [41] highlights the importance of conducting new epidemiological studies on hand injuries in the elderly population, especially those over 70 years old. The authors included four epidemiological studies in their review that provided extrapolated data on patients over 70 years old. However, the authors were only able to conclude that scaphoid fractures are rare but reported in the elderly population and that more epidemiological studies are required. They emphasized that the subject is often ignored in classical teaching, which could result in missed diagnoses.

Although not demonstrated by this study, it is valuable to integrate the results discussed above with the findings of Giustini et al. [1]. This study showed that patients over 80 years old tend to have longer hospital stays when hospitalized for upper extremity traumas as compared to younger patients. This is likely due to possible associated injuries that these patients may have, as demonstrated in the current study.

This epidemiological study, based on Emergency Department data, did not consider the outcomes of hand lesions among the elderly. This is an important issue to examine, given the fragility of these patients, whose mobility may already be limited. A rapid and full recovery is particularly important for the elderly, as hands are often necessary for using walking devices and maintaining some degree of autonomy.

The study has several limitations and biases. Firstly, the data were collected from ED clinical charts instead of being taken directly from the patients due to the retrospective nature of the study. Secondly, the severity of the lesions was evaluated using the triage color code, which is a subjective evaluation made by trained nurses and is biased due to associated injuries. Additionally, the assessment of indication to hand surgery was performed without taking into account limited indications resulting from comorbidities and lower functional requests. Lastly, data from the last five-year period might be underestimated due to the almost ten-month post-COVID-outbreak period, during which, the number of people visiting the ED decreased [29].

## 5. Conclusions

In conclusion, in addition to offering a database with numerous patients which can be useful to evaluate further changes in this kind of population, the authors believe that results from this study are helpful to better deal with elderly patients presenting at the ED with hand trauma as they might become more frequent. Particular attention should be paid to associated injuries and limiting indications to surgery when strictly necessary.

## Figures and Tables

**Table 1:** Demographics.

		2001–2020	Mean per Year
Total number of patients		991	50
Mean age of patients *		84.9	/
(range)		(80–100)	/
(Standard deviation)		(4.0)	/
Sex	Male	472 (47.6%)	24
	Female	519 (52.4%)	26

* in years.

**Table 2:** Type of injuries.

		2001–2020 (% of Total)	How Many Needed Surgery (and %)
Amputations and Sub-amputations		33 (3.3%)	30 (90.9%)
Fractures	Phalangeal	131 (13.2%)	50 (38.2%)
	Metacarpal	89 (9.0%)	46 (51.7%)
	Carpal	13 (1.3%)	0 (0%)
Dislocations		31 (3.1%)	15 (48.4%)
Tendinous (flexors and extensors)		27 (2.7%)	26 (96.3%)
Superficial Wounds		203 (20.5%)	30 (14.8%)
Bruises and Sprains		178 (18.0%)	0 (0%)
Pain		86 (8.7%)	0 (0%)
Infections		40 (4.0%)	25 (62.5%)
Others		160 (16.1%)	10 (6.3%)
TOTAL		991 (100%)	232 (23.4%)

## Data Availability

Data are available on demand by asking the corresponding author.
